# Spatio-Temporal Modelling Informing *Wolbachia* Replacement Releases in a Low Rainfall Climate

**DOI:** 10.3390/insects13100949

**Published:** 2022-10-18

**Authors:** Dan Pagendam, Samia Elfekih, Majed S. Nassar, Samuel Nelson, Abdulaziz M. Almalik, Essam A. Tawfik, Mohamed B. Al-Fageeh, Ary A. Hoffmann

**Affiliations:** 1 CSIRO Data61, Dutton Park, Brisbane, QLD 4101, Australia; 2 CSIRO H&B, Australian Centre for Disease Preparedness (ACDP), Geelong, VIC 3052, Australia; 3 Pest and Environmental Adaptation Research Group, Bio21 Institute and the School of Biosciences, University of Melbourne, Parkville, VIC 3052, Australia; 4 King Abdulaziz City for Science and Technology (KACST), Riyadh 11442, Saudi Arabia; 5 CSIRO Data61, Black Mountain, Canberra, ACT 2601, Australia

**Keywords:** *Aedes aegypti*, *Wolbachia*, pre-release, Saudi Arabia, arid, spatial modelling, ovitrap, population dynamics

## Abstract

**Simple Summary:**

*Aedes aegypti* is a mosquito that is responsible for spreading viral diseases including dengue fever, Zika, and chikungunya. Disease spread can be reduced by releasing mosquitoes containing bacteria known as *Wolbachia,* which inhibit transmission. Before such releases, it is important to collect data about where *Ae. aegypti* occur in urban landscapes and how populations vary over space and time. In this study, we present a pre-release analysis of mosquito populations using ovitraps (traps that provide a substrate for female mosquitoes to lay eggs), in Jeddah, Saudi Arabia. Our study contains a number of important findings. Firstly, we showed that there was no difference in the numbers of eggs laid between basement and non-basement locations. Secondly, we showed that, for some study sites, there was significant spatial structure to populations, meaning that where numbers of eggs were high (or low) they tended to remain high (or low) on average for many hundreds of meters. We also found that when mosquitoes are present in an area during the dry season, they tend to remain into the wet season; however, regions of high egg production could change between seasons. This suggests that in Jeddah’s arid environment, the quality of breeding environments is inconsistent over time.

**Abstract:**

Releases of *Aedes aegypti* carrying *Wolbachia* bacteria are known to suppress arbovirus transmission and reduce the incidence of vector-borne diseases. In planning for *Wolbachia* releases in the arid environment of Jeddah, Saudi Arabia, we collected entomological data with ovitraps across a 7-month period in four locations. Herein, we show that mosquito presence in basements does not differ from that of non-basement areas of buildings. In modelling mosquito presence across the study sites, we found the spatial structure to be statistically significant in one of the four sites, while a significant spatial structure was found for egg production data across three of the four sites. The length scales of the spatial covariance functions fitted to the egg production data ranged from 143 m to 574 m, indicating that high productivity regions can be extensive in size. Rank-correlation analyses indicated that mosquito presence tended to persist from the dry to wet season, but that egg production ranks at locations could reverse. The data suggest that, in Jeddah, the quality of the local environment for breeding can vary over time. The data support the feasibility of dry season releases but with release numbers needing to be flexible depending on local rates of invasion.

## 1. Introduction

Releases have been undertaken with *Wolbachia*-infected mosquitoes around the world to suppress the transmission of arboviruses spread by *Aedes aegypti* mosquitoes, and to reduce the burden of vector-borne diseases such as dengue fever, chigungunya, and Zika. Previous release operations took place in humid tropical areas such as northern Australia [1], Indonesia [2], Vietnam [3], and Malaysia [4]. Many of these releases are targeted at replacing the existing mosquito populations with ones that are infected by a new *Wolbachia* strain (“replacement”) and show lower disease transmission, with impressive field results so far [4,5]. However, release trial results have not yet been reported in hot, low rainfall climates in which *Ae. aegypti* can occur along with substantial arbovirus transmission [6,7].

Populations in such environments provide novel challenges for releases. *Ae. aegypti* mosquitoes can persist through unfavorable conditions by entering quiescence or by becoming restricted to favorable areas within human habitations [8]. Breeding sites within buildings and in large storage and subterranean water tanks can be particularly important during unfavorable seasons [9,10,11]. However, many breeding sites and larval habitats are likely to remain cryptic and this can mean that attempts to control mosquitoes through multiple and repetitive chemical applications and through targeting breeding sites often fail (e.g., [11,12]).

One of the features of *Ae. aegypti* outbreaks is that the numbers of mosquitoes can vary spatially at quite a fine scale. Typically, there are local areas where mosquitoes are abundant, interspersed with other areas where numbers are lower at fine spatial scales [13]. Local high-density areas may be identifiable through specific environmental factors to some extent (e.g., [4,14,15,16]). Trap catches can be relatively stable across time in some areas [17], but this is not always the case with high mosquito counts for a period in one area being replaced by other local areas even within the same village or suburb. This type of local heterogeneity can influence effective treatments, including targeted chemical spraying [18], but also influences releases of *Wolbachia*-infected mosquitoes aimed at population suppression [19].

The city of Jeddah, situated in the western part of Saudi Arabia, is a coastal port city on the red Sea, known for its hot, humid, and low-rainfall climate (BWh; Hot Desert Climate) under the Koppen’s classification [20]. In Jeddah, there is a continuously detectable adult population of *Ae. aegypti* [21,22] although adult numbers are thought to increase in the cooler and wetter periods of December–February. Since 1993, mosquito populations have been linked to dengue fever outbreaks in Jeddah. Since then, dengue cases have been reported in Jeddah and other neighboring regions in Saudi Arabia. The burden of dengue fever in the Kingdom has been estimated at USD 117M/year [23]. Mosquito vectors are mostly suppressed through pesticides but these are not necessarily successful [24] and there are increasing problems associated with resistance, particularly to pyrethroids [25,26,27,28]. The presence of adults all year round suggests that mosquitoes are most likely using favorable sites given that outside temperatures during the hot dry season are extremely high, with a maximum average daily temperature in the hottest months exceeding 36 °C. However, while adults can be caught all year, there may be a substantial contribution of adults developing from quiescent egg banks in favorable wet periods.

The relative importance of ongoing breeding versus mosquitoes from egg banks and local heterogeneity in mosquito numbers are important because they affect the outcome of *Wolbachia*-based release strategies, directly and indirectly, through density-dependent processes [29]. Large egg bank contributions would mean that local invasion in some areas could be overwhelmed in a short, wet season when many eggs hatch and the local density in some habitats will be high. These factors would tend to favor releases after mosquitoes from the egg bank have hatched, particularly when *Wolbachia*-infected strains have lower hatch rates from long-stored eggs and also suffer from a decrease in fecundity when hatched from these eggs [30,31,32]. On the other hand, if, after the dry season, most mosquitoes come from populations that have continued to persist and develop in refugial areas, then *Wolbachia* invasions may be easier in the middle or the end of the dry season when natural population sizes are low and fewer mosquitoes have to be released to achieve replacement.

In tracking *Wolbachia* invasion across an area, it is necessary to implement a rapid method of spatially assessing mosquito numbers in some detail so that problematical areas for invasion can be rapidly identified. This requires an approach in which large numbers of traps can be deployed without the need for detailed house inspections. *Wolbachia* interventions, therefore, implement various types of ovitraps or gravitraps for rapid monitoring. To investigate the feasibility of such an approach for tracking mosquito numbers in detail across an area, we collected entomological data from multiple sites in Jeddah using simple ovitraps and tracked mosquito numbers across space moving from the wet season to the dry period. Our main aims were: (1) to investigate if there is spatial consistency in mosquito numbers across this period—as might be expected if breeding remains confined to specific sites and local regions; (2) to measure the extent of the decrease in mosquito numbers in multiple sites as we move from the wet season to the dry season; and (3) to compare results from ovitraps placed in shaded and accessible areas to previous results with different trapping and surveying approaches implemented in Jeddah.

## 2. Materials and Methods

### 2.1. Data Collection

**Figure 1:**
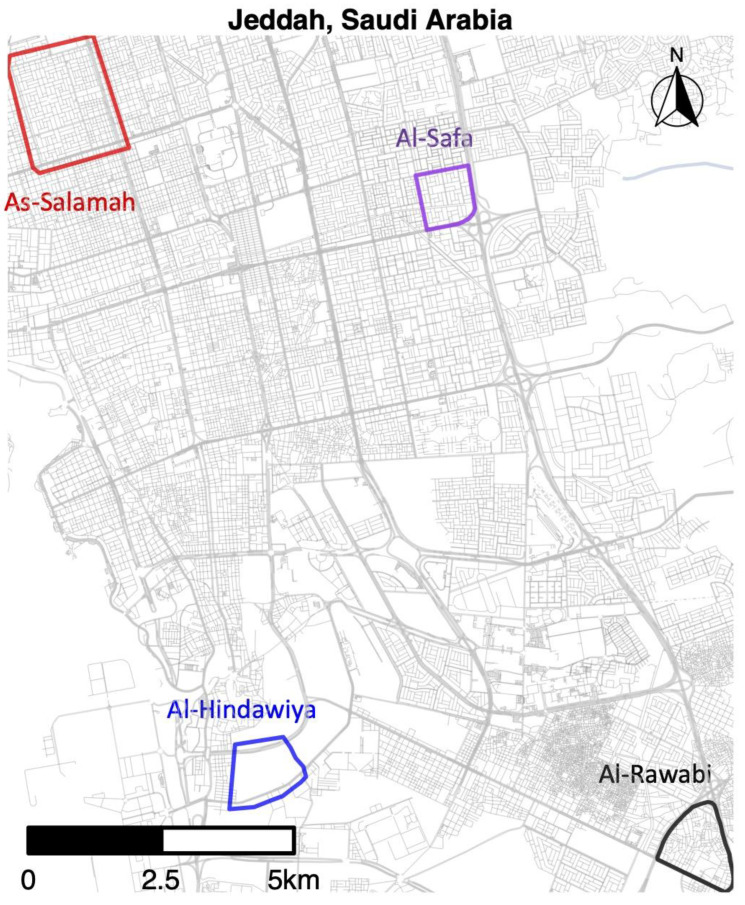
Map of Jeddah (western Saudi Arabia) showing the geographical location of the four districts where pre-release sampling occurred from December 2020 to April 2022.

We monitored mosquito populations in four regions of Jeddah, Saudi Arabia, over the period spanning from 29 December, 2020 to 20 April 2022. The study sites were located in the suburbs of As-Salamah, Al-Safa, Al-Hindawiya, and Al-Rawabi; the locations of these study areas across Jeddah are shown in Figure 1. Al-Safa, Al-Hindawiya, and Al-Rawabi were monitored over the period 29 December 2020–1 June 2021, whilst monitoring of As-Salamah continued up to 20 April 2022. In each region, sampling of the mosquito population was undertaken using ovitraps, each consisting of a red felt attached to a black, waterfilled bucket (1.25 L capacity; 142 mm top diameter; 110 mm bottom diameter) containing approximately 200–250 mL of water and 2–3 grass pellets (purchased from a local farm supplier in Jeddah), which provide an attractive substrate for female *Ae. aegypti* egg laying. These traps have been used previously in Jeddah for a movement study [33] and also in other locations.

Traps were placed in shaded areas in the basements and other parts of buildings, near the main entrance, under the stairs, and behind small shrubs in front of houses and duplex buildings. The placement of the ovitraps in these particular locations was decided based on whether field workers were able to access the buildings, both for the placement and collection of the traps, at the end of the sampling operation. Mosquito diversity in Jeddah is very low, and *Ae. aegypti* is the most prevalent species in the field by far, comprising over 96% of trapped mosquitoes [34], so egg counts were used to investigate the *Ae. aegypti* population size in the districts of interest.

Ovitraps were left in the field for one week at a time before the felts were collected from them and returned to the laboratory. To ensure proper and efficient data management, we used Open Data Kit (ODK) Central as a cloud-based data repository [35]. We created customized data collection forms that were developed specifically for the Jeddah *Wolbachia* project, for use with the ODK Collect (https://github.com/getodk/collect, access on 1 March 2021) application on Android OS-powered mobile devices. These forms were used by field specialists to record GPS locations, timestamps, and to scan unique QR codes for each felt specimen collected from the field. Felts were then brought to the laboratory, where they were processed by the technicians using the ODK Collect app to scan the same QR code (attached to the sample) and record the number of *Ae. aegypti* eggs (identified by visual inspection under microscope) that were present on each felt. Field and laboratory data were extracted from ODK Central using R [36] and the R package “ruODK” [37]. The data were then joined using the unique QR codes attached to felts so that spatial and temporal trends in mosquito presence/absence and mosquito productivity (numbers of eggs laid) could be analyzed.

**Figure 2:**
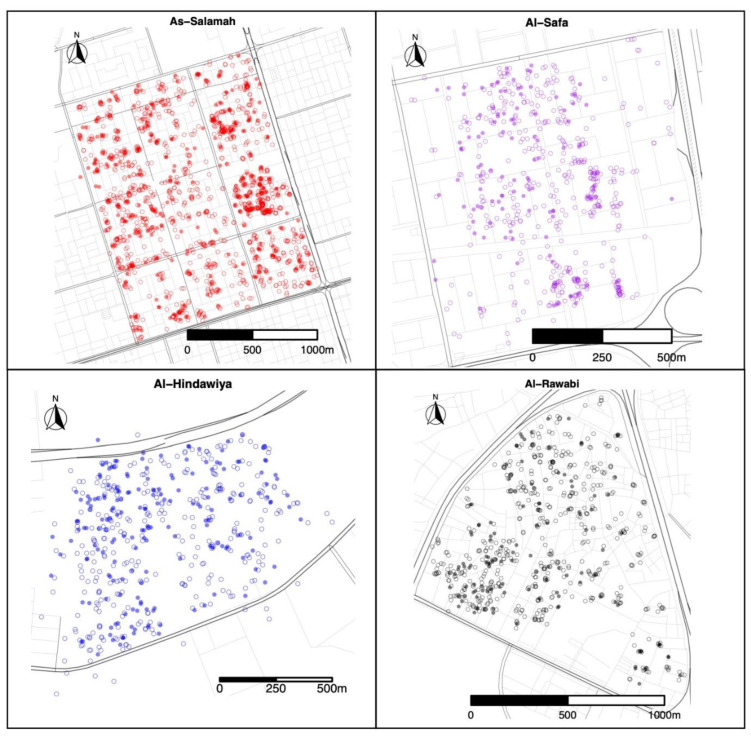
Spatial locations of felt collections over the four districts (As-Salamah, Al-Safa, Al-Hindawiya, and Al-Rawabi) in Jeddah, Saudi Arabia, across all data collection periods (from December 2020 to April 2022). Filled and non-filled circles show felts that were positive and negative for presence of *Ae. aegypti* mosquito eggs, respectively.

**Figure 3:**
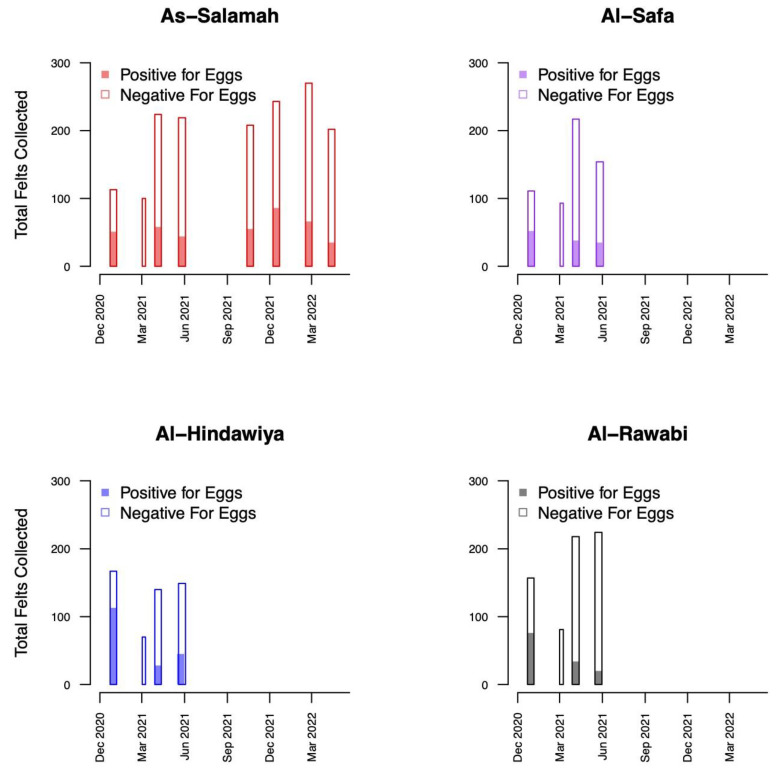
Bar plots showing the temporal distribution of felt collections over the four districts (As-Salamah, Al-Safa, Al-Hindawiya, and Al-Rawabi) in Jeddah, Saudi Arabia. Bar widths correspond to the intervals (either one or two weeks) over which felt collections were performed. Colored and non-colored proportions of bars show the proportions of collected felts that were positive and negative for egg presence, respectively.

A total of 1579 felts were collected (395 with eggs) in As-Salamah, 575 in Al-Safa (126 with eggs), 526 in Al-Hindawiya (187 with eggs), and 680 in Al-Rawabi (130 with eggs). The spatial and temporal distributions of all felts and the presence/absence of eggs on felts across the four regions are shown in Figure 2 and Figure 3, respectively. Figure 3 shows how data collection was achieved over a two-week period: it commenced with trap deployment; this was followed by the first felt collection; then second felt deployment occurred after 7 days, and we finished with the second felt collection after 14 days. The exception to this was the field sampling performed in March 2021, which only included one felt collection, with only a few felts registering as positive for egg presence.

### 2.2. Data Analysis

As a first step, exploratory data analyses were undertaken to study how mosquito presence and egg production varied with different explanatory variables, namely, (i) whether the ovitrap was deployed above ground or in a basement location; (ii) the month of the year and associated meteorological variables; and (iii) site and geographical location.

Following this, we used the “brms” package [38] for R (R Core Team, 2021) to undertake Bayesian statistical analyses. Each analysis was performed using four independent Markov chains, each consisting of 5000 iterations for warmup and a further 5000 iterations for inference. The sampling algorithm chosen for this purpose was the “No U-Turn Sampler” [39].

Firstly, we used the “brms” R package to study whether the placement of traps in basements had a statistically significant effect on the presence of eggs and on the number of eggs per felt. For the presence of eggs, we fitted a generalized linear model (GLM) with a Bernoulli response variable and logit-link function for the mean Group-level random intercepts were included for each collection event and the population-level intercept and basement effect. For the number of eggs per felt, we used the same model structure, but used a zero-inflated negative-binomial data model with a log-link function for the negative binomial mean and shape parameter and a logit-link function for the zero-inflation parameter. A zero-inflated data model was used since many collected felts had no eggs and the model allowed the level of zero-inflation to vary as a logit-function of the basement effect and indicator variables for collection event. Prior distributions for all parameters in the models were chosen to be uninformative (the default values in “brms”).

Secondly, we used the “brms” package for R to build statistical models to examine whether there was a significant elevated presence of mosquito eggs and egg production on ovitrap felts collected in December and January compared to the rest of the year. We fitted generalized linear models with a Bernoulli response variable (logit-link function for the mean) for egg presence and a zero-inflated negative-binomial data model, with the same families of link functions described previously, for the number of eggs per felt. In both models, we used an indicator variable for the season (January and December versus other) and group-level random intercepts for each felt collection and each study region. The same model structure mentioned previously was used for modelling the zero-inflation in the data. As before, prior distributions for all parameters in the models were chosen to be uninformative (the default values in “brms”).

Thirdly, we fit spatial statistical models to mosquito presence and egg production data collected in each of the regions using the “brms” R package. For each region, a spatial Gaussian process (i.e., kriging) model was fit to the data within unique December–January and February–November intervals. For As-Salamah, this resulted in four Gaussian process models for each response variable, since data were collected over two years. For the other three sites, two Gaussian processes were fit for each of the response variables.

All Gaussian process models used an isotropic, squared-exponential covariance kernel. For each analysis, we compared the spatial model to a simpler null model that simply used a spatially homogeneous mean for each response variable. A comparison was made by estimating the difference in the expected log predictive density (ELPD) metric [40] between the Gaussian process model and the null model. The standard error of this difference was also estimated. Using this, we then used a simple z-test (in general, the assumption of normality is considered valid when the ELPD difference > 4) to ascertain if the ELPD for the Gaussian process model was significantly greater than that of the null model. The estimated length-scale parameter from each Gaussian process model fit to the data was also extracted from the statistical model to provide an indication of what range spatial correlation occurred over. Under the squared-exponential covariance model with a length-scale of L meters, two locations separated by L meters would have a correlation of 0.61, and this diminishes to a correlation of 0.14 at a distance of 2 L meters.

After fitting the predicted surfaces from spatial statistical models, we also computed rank correlations [41] between temporally consecutive spatial fields using their values at sampling locations (where spatial predictions have higher precision). The pointwise rank correlations were intended to provide an indication of whether the predicted spatial fields showed evidence of ordinal association (i.e., whether regions with high densities of mosquitoes persisted or not).

We generated contingency tables to test for dependence between egg presence/absence on felts collected at co-located traps on: (i) consecutive sampling events (seven days apart); and (ii) collections made 30–90 days apart. Due to low counts in some cells, Fisher’s exact test was used to test the null hypothesis that no temporal dependence in mosquito presence existed.

## 3. Results

**Figure 4:**
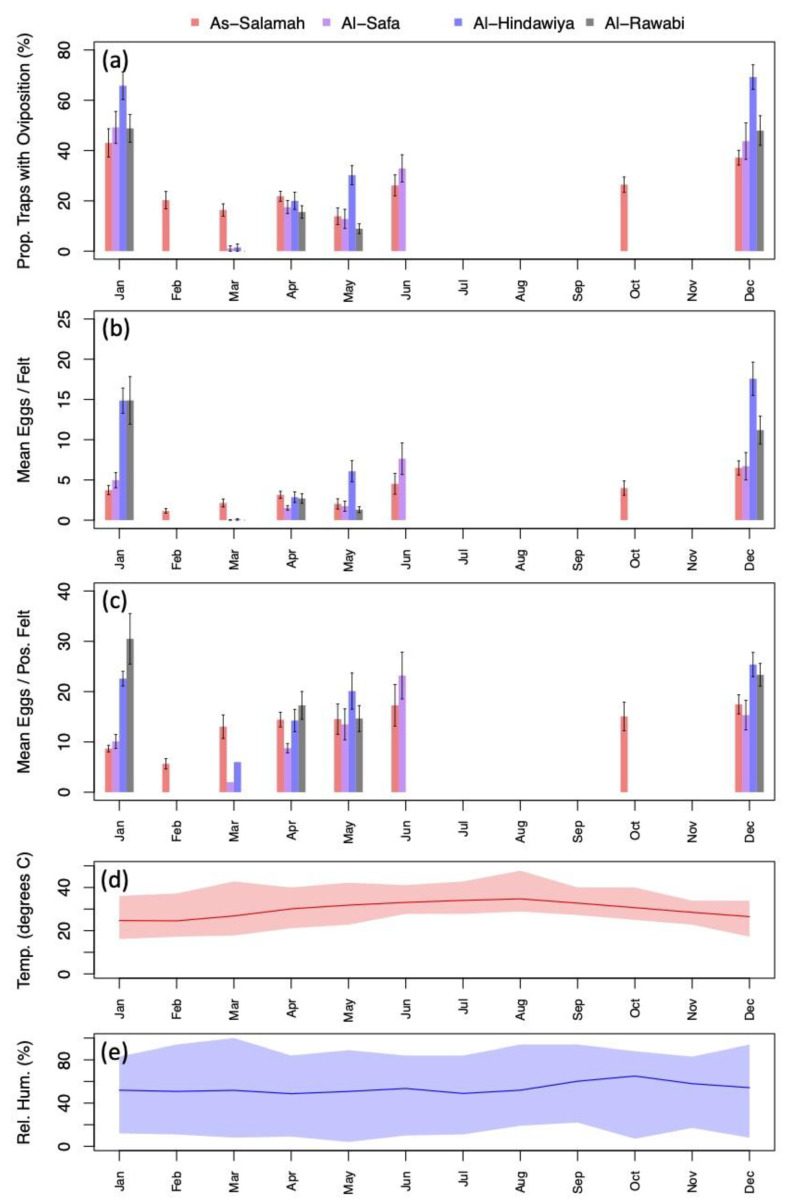
(**a**) Percentage of traps with oviposition by month (aggregated across study sites); (**b**) mean number of eggs deposited per felt by month (aggregated across sites); (**c**) mean number of eggs deposited per positive felt by month (aggregated across sites); (**d**) mean monthly temperature (line) with monthly minimums and maximums spanned by colored regions; (**e**) mean monthly relative humidity (line) with monthly minimums and maximums spanned by colored regions. Black intervals extending from barplots in (**a**–**c**) show ± one standard error.

**Figure A1:**
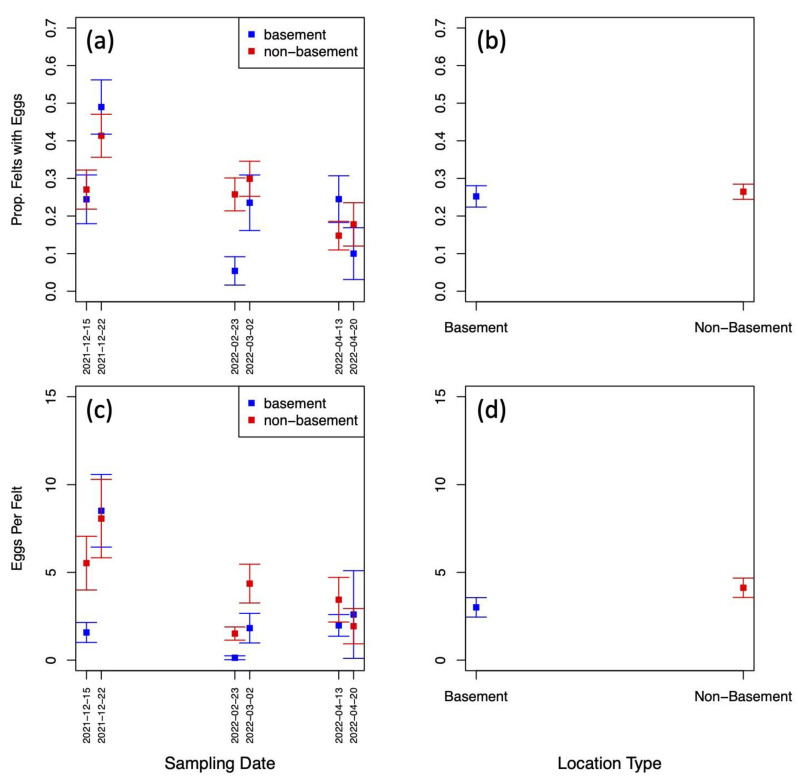
(**a**) Proportion of egg-positive felts collected from basement and non-basement locations in As-Salamah plotted by collection date; (**b**) Proportion of egg-positive felts collected from basement and non-basement locations in As-Salamah, aggregated over all collection dates; (**c**) mean numbers of eggs on felts collected from basement and non-basement locations in As-Salamah plotted by collection date; (**d**) mean numbers of eggs on felts collected from basement and non-basement locations in As-Salamah, aggregated over all collection dates; Bars extending from plotted squares show the standard errors of the estimates.

**Figure A2:**
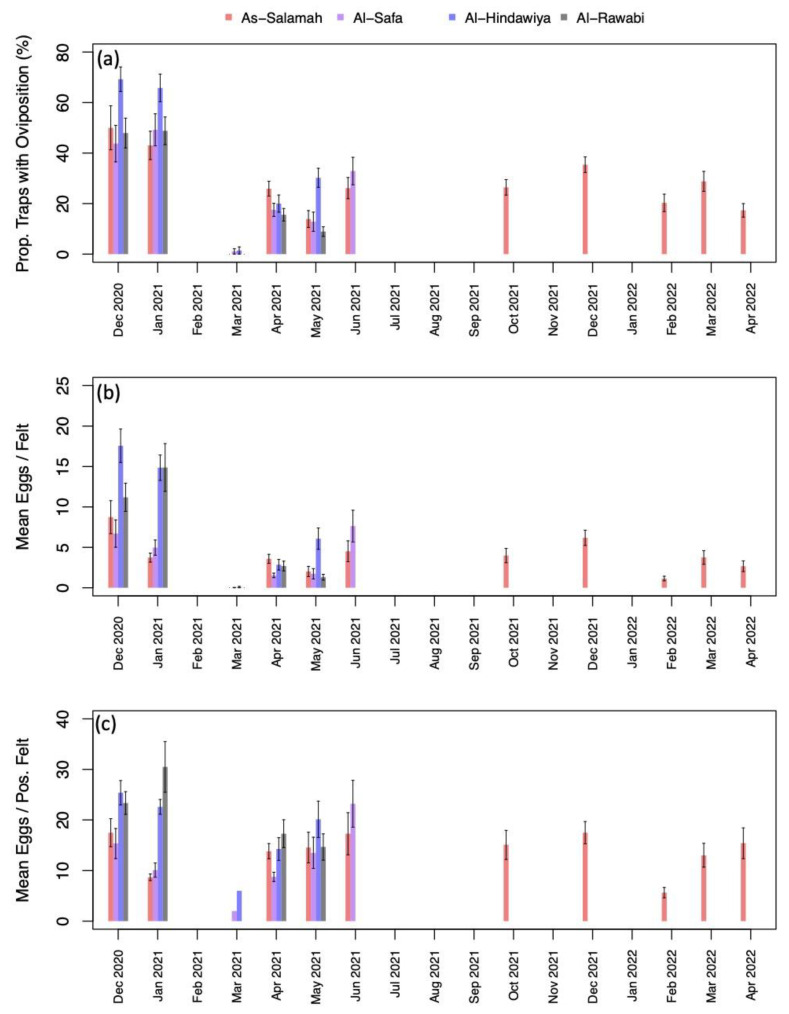
(**a**) percentage of traps with oviposition by month and year (aggregated across study sites); (**b**) mean number of eggs deposited per felt by month and year (aggregated across sites); and (**c**) mean number of eggs deposited per positive felt by month and year (aggregated across sites). Black intervals extending from barplots in (**a**–**c**) show +/– one standard error.

Exploratory data analysis of ovitrap data collected from the four selected districts in Jeddah showed the spatial sampling of the collected data, with a few gaps linked to schools, mosques, parks, and shopping centers that could not be sampled, and with positive traps collected throughout the area (see Figure 2). Plots of the proportion of egg-positive collected felts and the number of eggs per felt, by month, indicated that both response variables tended to be higher (on average) in December and January compared to other months (see Figure 4 and Figure A2). In contrast, the numbers of eggs laid per egg-positive felt appeared to remain relatively consistent across months, and for a few sites, the highest numbers were recorded in the June collection (Figure 4). Data from As-Salamah, where information related to basement versus non-basement sites was available, were plotted separately for these locations in Figure A1 of the Appendix A and did not reveal obvious differences in averages at each sampling date or when averaged over all sampling dates.

Statistical modelling of the data showed that there was no significant difference in the presence/absence of mosquito eggs on ovitrap felts collected from basement and non-basement locations (the Bayesian 95% credible interval for basement effect contains zero; see Appendix A Table A1). However, the analysis of egg production did reveal evidence of mean numbers of eggs per collected felt being lower for basement locations compared to non-basement locations (the Bayesian 95% credible interval for basement effect spans negative values only; see Appendix A Table A2). Furthermore, there did not appear to be any strong evidence of a difference in the zero-inflation of the egg numbers between basement and non-basement locations. Figure A1d of the Appendix A clearly demonstrates that whilst a difference in mean eggs per felt between basement and non-basement locations might exist, the effect size is small.

Contingency tables for dependence between egg presence/absence at co-located basement traps over different time periods are provided in Appendix A Table A3 and Table A4. For co-located traps separated by a week and also by 30–90 days, Fisher’s exact test suggested no evidence of dependence (*p* = 1 and *p* = 0.35, respectively). These data suggest that basements that were positive at one time point did not tend to be positive at a later time point.

Further modelling showed the statistical significance of higher mosquito presence and egg production in January and December compared to other times of the year. For mosquito presence, the model coefficients for the period February to November had Bayesian 95% credible intervals that completely spanned negative ranges of values (i.e., did not include zero), providing strong evidence for a significant difference between the two periods (see Appendix A Table A5). For eggs laid per felt, there was strong evidence of an increase in zero-inflation between February and November compared to January and December; however, there was no evidence of the negative binomial distribution mean being significantly different between these seasons after accounting for zero-inflation (see Appendix A Table A6).

Spatial statistical models for mosquito presence showed that the spatial heterogeneity inferred through the inclusion of the Gaussian process could be considered statistically significant (*p* < 0.05) for Al-Hindawiya (*p* = 0.002), but not for Al-Rawabi (*p* = 0.058), Al-Safa (*p* = 0.124), or As-Salamah (*p* = 0.159). The length scales of Gaussian process covariance kernels were estimated to be similar across all regions and are listed in Table 1.

Conversely, spatial statistical models for the number of eggs per felt showed that the spatial heterogeneity inferred through the inclusion of the Gaussian process could be considered statistically significant (*p* < 0.05) for Al-Rawabi (*p* = 0.004), Al-Safa (*p* = 0.049), and As-Salamah (*p* = 0.031), but not for Al-Hindawiya (*p* = 0.057). The length scales of Gaussian process covariance kernels were estimated to be similar across all sampling regions and are listed in Table 2.

**Figure 5:**
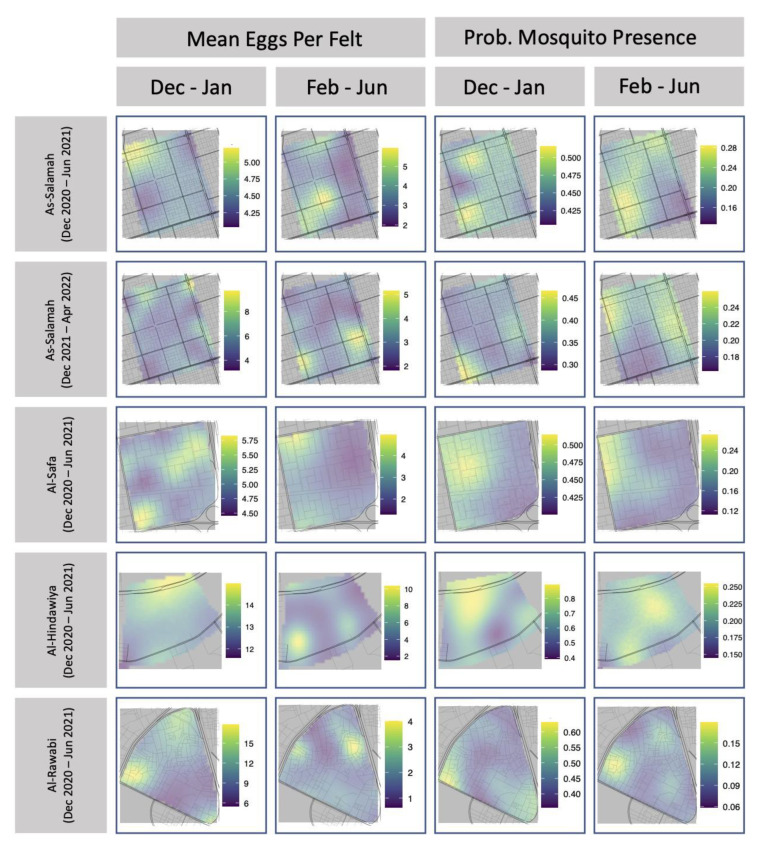
Estimated spatial fields for mean number of eggs per felt and probability of mosquito presence for ovitrap felts collected in each of the four study areas.

Tables of Kendall’s rank correlation are provided in Appendix A Table A7 and Table A8. For mosquito presence, rank correlations with larger magnitudes tended to be positive, but the value with the largest magnitude was only 0.39. This indicated that temporally consecutive spatial fields of mosquito presence showed some weak signs of retaining an ordinal association over time (i.e., that mosquito presence persists over time). Conversely, for mosquito egg production, rank correlations with larger magnitudes tended to be negative, but the value with the largest magnitude was only −0.34. This indicated that temporally consecutive spatial fields of mean egg production showed some weak evidence of having a reversed ordinal association. In other words, locations that were ranked as high egg production locations in one spatial field, tended to be of lower rank in the subsequent spatial field. Subtle evidence of these patterns can be observed in Figure 5, which maps the predicted spatial fields for each collection period in each region.

## 4. Discussion

The pre-release data confirm the presence of active *Ae. aegypti* throughout the year in Jeddah at all four sites as detected by the ovitraps. These simple traps are cheap, simple to construct, and easy to deploy in their hundreds. The higher incidence of positive traps at two sites in January and December is consistent with a more active mosquito population in those months, coinciding with periods of rain and cooler climate conditions. However, the mosquitoes clearly remain active in some areas at other times, despite the high temperatures and lack of rain, as noted in previous studies using different data sources, such as light traps and larval surveys (e.g., [22,42]). At this time, we found *Ae. aegypti* breeding in a variety of areas in buildings in which water accumulates due to poor drainage (e.g., car parking areas and blocked drains). Previous surveys noted breeding sites such as drainage holes and various water storage containers [43,44].

From the perspective of releases, these data suggest that releases with *Wolbachia*-infected mosquitoes could be undertaken at any time of the year. If there is a steady turnover of mosquitoes across the year, it may be beneficial to release adults in the dry season when population numbers are low and therefore invasion is easier, given that this depends on exceeding an invasion point above which the *Wolbachia* will spread and then remain stable at a high frequency in a natural population [1,4]. On the other hand, it is still possible that some eggs persist in areas through a quiescent phase, which can last for several months [18] and make *Wolbachia* invasion challenging due to the costs associated with egg quiescence [32]. Some eggs might only hatch in the rainy season. However, we note in our surveys that the number of eggs on felts, where present, can be quite high even in the extended dry season. This suggests that local abundance of mosquitoes can be quite high in sites even if there are fewer suitable sites.

The ovitrap data provide an unprecedented picture of the distribution of breeding *Ae. aegypti* across the study areas and, in each case, there seems to be a fairly even distribution of positive traps across the sampled areas where traps could be placed. This pattern strongly suggests a widespread mosquito population that is not restricted to a few “hot spots” within an area. Nevertheless, we also established the spatial structure for egg numbers on felts and (in one case) the presence of mosquitoes, suggesting that *Wolbachia* invasion dynamics following release may be heterogeneous. Such fine scale heterogeneity has been established in previous releases [19]. The spatial distance involved in the kernels of several hundred meters fits well with estimated inter-generational movement distances of around 80–200 m estimated from Al-Safa district in Jeddah [33].

The results also point to a lack of consistent mosquito “hot spots” in the monitored regions. If persistent areas of high mosquito density persisted, we might have expected a strong spatial correlation when comparing felt egg counts across time, and we might have also expected to identify basements that continued to produce large numbers of mosquitoes. Instead, it appears that local areas with high *Ae. aegypti* populations vary over months, making it critical to release widely when undertaking *Wolbachia* interventions. Modelling has suggested that releases in spatially or temporally heterogeneous areas may slow invasion rates if there are fitness costs associated with the *Wolbachia* [29]. This may make invasion by the *w*AlbB *Wolbachia* strain more difficult than *w*Mel, which suffers from smaller fitness costs, although other factors such as the stability of the *Wolbachia* infection under heat [45] will also be particularly important within the Jeddah *Wolbachia* releases context. The pre-release sampling and data modelling not only assists local stakeholders in deciding on the optimal approach for releasing mosquitoes under a *Wolbachia* replacement approach, but also provides information relevant to a more comprehensive integrated dengue management strategy that interfaces with the *Wolbachia* approach and involves the elimination of breeding sources and larval habitats using targeted larvicide treatments.

## 5. Conclusions

In summary, we used low-cost traps to show the widespread distribution of mosquitoes across the sampling areas with some evidence for a spatial structure that did not necessarily persist across time. These patterns point to the suitability of a release strategy across the area, with the possibility of releases being initiated in the drier season; however, heterogeneous mosquito densities potentially make *Wolbachia* invasion more challenging as a result of the substantial fitness costs.

## Figures and Tables

**Table 1:** Estimated length-scale parameters of covariance kernels in Gaussian process models for mosquito presence.

District	Field Work Period	Estimated Length-Scale (m)	Std. Err. (m)	95% Credible Interval (m)
Al-Hindawiya	December 2020–January 2021	302	137	[109, 660]
Al-Hindawiya	February 2021–June 2021	330	219	[27, 851]
Al-Rawabi	December 2020–January 2021	287	172	[57, 745]
Al-Rawabi	February 2021–June 2021	229	143	[29, 573]
Al-Safa	December 2020–January 2021	376	211	[24, 822]
Al-Safa	February 2021–June 2021	423	211	[47, 846]
As-Salamah	December 2020–January 2021	485	353	[4, 1280]
As-Salamah	February 2021–June 2021	795	353	[88, 1545]
As-Salamah	December 2021–January 2022	573	397	[44, 1324]
As-Salamah	February 2022–April 2022	706	353	[44, 1457]

**Table 2:** Estimated length-scale parameters of covariance kernels in Gaussian process models for mean mosquito egg number per felt.

District	Field Work Period	Estimated Length-Scale (m)	Std. Err. (m)	95% Credible Interval (m)
Al-Hindawiya	December 2020–January 2021	385	247	[0, 907]
Al-Hindawiya	February 2021–June 2021	247	82	[54, 357]
Al-Rawabi	December 2020–January 2021	401	172	[86, 745]
Al-Rawabi	February 2021–June 2021	143	86	[57, 3344]
Al-Safa	December 2020–January 2021	212	165	[0, 658]
Al-Safa	February 2021–June 2021	423	165	[94, 752]
As-Salamah	December 2020–January 2021	574	397	[0, 1413]
As-Salamah	February 2021–June 2021	265	265	[132, 1192]
As-Salamah	December 2021–January 2022	309	265	[44, 1104]
As-Salamah	February 2022–April 2022	265	265	[88, 1060]

## Data Availability

R scripts and data for replicating these analyses are available at the CSIRO Data Access Portal via the following entry link: https://data.csiro.au/collection/csiro:55849.

## Appendix A

**Table A1:** Summary of Bayesian generalised linear model to assess the significance of basement versus non-basement ovitrap locations on mosquito egg presence (Bernoulli response variable, log-link function). There were six collection events which were treated as group-level effects that contributed an intercept. Population-level effects were an intercept and the effect of the basement variable (i.e., whether the trap was placed in a basement). Columns for effects and estimated parameters are: posterior mean estimate (“Estimate”); standard error of the posterior mean (“Est. Err.”); lower limit of the 95% Bayesian credible interval (“Lower 95%”); upper limit of the 95% Bayesian credible interval (“Upper 95%”); Gelman-Rubin convergence statistic (“Rhat”); bulk effective sample size for the estimate (“Bulk ESS”); and the tail effective sample size of the estimate (“Tail ESS”).

**Group-Level Effects**							
**Collection Instance (Number of levels: 6)**							
	**Estimate**	**Est. Err.**	**Lower 95%**	**Upper 95%**	**Rhat**	**Bulk ESS**	**Tail ESS**
sd(Intercept)	0.66	0.32	0.27	1.51	1.00	4563	6396
**Population-Level Effects**							
	**Estimate**	**Est. Err.**	**Lower 95%**	**Upper 95%**	**Rhat**	**Bulk ESS**	**Tail ESS**
Intercept	–1.06	0.31	–1.69	–0.42	1.00	5119	6860
Basement	–0.12	0.19	–0.50	0.25	1.00	13,715	12,280

**Table A2:** Summary of Bayesian generalised linear model to assess the significance of basement versus non-basement ovitrap locations on mosquito eggs laid per felt. A model was fit for the mean of the negative-binomial distribution (log-link function) and simultaneously to the zero-inflation (Z.I.) probability (logit-link function). There were six collection events which were treated as group-level effects that contributed intercepts to the two models. Population-level effects were an intercept and the effect of the basement variable (i.e., whether the trap was placed in a basement) in both models. Columns for effects and estimated parameters are: posterior mean estimate (“Estimate”); standard error of the posterior mean (“Est. Err.”); lower limit of the 95% Bayesian credible interval (“Lower 95%”); upper limit of the 95% Bayesian credible interval (“Upper 95%”); Gelman-Rubin convergence statistic (“Rhat”); bulk effective sample size for the estimate (“Bulk ESS”); and the tail effective sample size of the estimate (“Tail ESS”).

**Group-Level Effects**							
**Collection Instance (Number of levels: 6)**							
	**Estimate**	**Est. Err.**	**Lower 95%**	**Upper 95%**	**Rhat**	**Bulk ESS**	**Tail ESS**
sd(Intercept)	0.73	0.37	0.29	1.68	1.00	5629	7332
sd(Intercept Z.I.)	0.65	0.33	0.25	1.50	1.00	6214	9045
**Population-Level Effects**							
	**Estimate**	**Est. Err.**	**Lower 95%**	**Upper 95%**	**Rhat**	**Bulk ESS**	**Tail ESS**
Intercept	2.54	0.35	1.79	3.20	1.00	6496	7484
Basement	–0.57	0.20	–0.97	–0.18	1.00	20,900	14,381
Intercept Z.I.	0.87	0.31	0.22	1.49	1.00	7002	8210
Basement Z.I.	0.03	0.21	–0.38	0.43	1.00	24,291	13,717
**Estimated Parameters of Zero-Inflated Negative Binomial Data Model**							
	**Estimate**	**Est. Err.**	**Lower 95%**	**Upper 95%**	**Rhat**	**Bulk ESS**	**Tail ESS**
Neg.-Bin. Shape	0.81	0.14	0.55	1.11	1.00	20,460	13,233

**Table A3:** Contingency tables for egg presence (+) or absence (–) at co-located basement traps sampled 7 days apart.

7 Days Apart		
	Second Felt +	Second Felt –
First Felt +	2	4
First Felt –	12	27

**Table A4:** Contingency tables for egg presence (+) or absence (–) at co-located basement traps sampled 30–90 days apart.

30–90 Days Apart		
	Second Felt +	Second Felt –
First Felt +	3	24
First Felt –	10	37

**Table A5:** Summary of Bayesian generalised linear model to assess the significance of season on mosquito egg presence (Bernoulli response variable, log-link function). Ovitrap felt collections were grouped into 15 events over the four study areas, each of which were modelled using a random intercept. Population-level effects were an intercept and the effect of a binary variable for season (0: December and January; 1: February to November). Columns for effects and estimated parameters are: posterior mean estimate (“Estimate”); standard error of the posterior mean (“Est. Err.”); lower limit of the 95% Bayesian credible interval (“Lower 95%”); upper limit of the 95% Bayesian credible interval (“Upper 95%”); Gelman-Rubin convergence statistic (“Rhat”); bulk effective sample size for the estimate (“Bulk ESS”); and the tail effective sample size of the estimate (“Tail ESS”).

**Group-Level Effects**							
**Collection Instance (Number of levels: 16)**							
	**Estimate**	**Est. Err.**	**Lower 95%**	**Upper 95%**	**Rhat**	**Bulk ESS**	**Tail ESS**
sd(Intercept)	0.97	0.24	0.61	1.54	1.00	4982	8621
**Collection Region (Number of levels: 4)**							
	**Estimate**	**Est. Err.**	**Lower 95%**	**Upper 95%**	**Rhat**	**Bulk ESS**	**Tail ESS**
sd(Intercept)	0.61	0.42	0.20	1.79	1.00	5793	7604
**Population-Level Effects**							
	**Estimate**	**Est. Err.**	**Lower 95%**	**Upper 95%**	**Rhat**	**Bulk ESS**	**Tail ESS**
Intercept	–0.21	0.60	–1.38	1.0	1.00	6305	7352
Feb. to Jun.	–1.32	0.58	–2.47	–0.17	1.00	5591	7752

**Table A6:** Summary of Bayesian generalised linear model to assess the significance of season on mosquito eggs laid per felt. A model was fit to the mean of the negative-binomial response variable (log-link function) and simultaneously to the zero-inflation (Z.I.) probability (logit-link function). Ovitrap felt collections were grouped into 15 events over the four study areas, each of which contributed a random intercept to both models. Population-level effects were an intercept and the effect of a binary variable for season (0: December and January; 1: February to November) in both models. Columns for effects and estimated parameters are posterior mean estimate (“Estimate”); standard error of the posterior mean (“Est. Err.”); lower limit of the 95% Bayesian credible interval (“Lower 95%”); upper limit of the 95% Bayesian credible interval (“Upper 95%”); Gelman-Rubin convergence statistic (“Rhat”); bulk effective sample size for the estimate (“Bulk ESS”); and the tail effective sample size of the estimate (“Tail ESS”).

**Group-Level Effects**							
**ollection Instance (Number of levels: 16)**							
	**Estimate**	**Est. Err.**	**Lower 95%**	**Upper 95%**	**Rhat**	**Bulk ESS**	**Tail ESS**
sd(Intercept)	0.39	0.11	0.22	0.66	1.00	5873	9874
sd(Intercept Z.I.)	0.96	0.24	0.61	1.53	1.00	6315	10,041
**Collection Region (Number of levels: 4)**							
	**Estimate**	**Est. Err.**	**Lower 95%**	**Upper 95%**	**Rhat**	**Bulk ESS**	**Tail ESS**
sd(Intercept)	0.58	0.44	0.19	1.77	1.00	5483	6611
sd(Intercept Z.I.)	0.62	0.44	0.20	1.81	1.00	6917	9386
**Population-Level Effects**							
	**Estimate**	**Est. Err.**	**Lower 95%**	**Upper 95%**	**Rhat**	**Bulk ESS**	**Tail ESS**
Intercept	2.89	0.43	2.01	3.66	1.00	7110	6032
Feb. to Jun.	–0.27	0.25	–0.77	0.21	1.00	8475	9912
Intercept Z.I.	0.12	0.62	–1.13	1.30	1.00	7664	9400
Feb. to Jun. Z.I.	1.33	0.58	0.18	2.51	1.00	7311	9919
**Estimated Parameters of Zero-Inflated Negative Binomial Data Model**							
	**Estimate**	**Est. Err.**	**Lower 95%**	**Upper 95%**	**Rhat**	**Bulk ESS**	**Tail ESS**
Neg.-Bin. Shape	1.39	0.09	1.22	1.58	1.00	23,281	13,513

**Table A7:** Correlation in modelled mosquito presence surfaces for trap collection locations.

District	Comparison	Kendall’s Rank Correlation
Al-Hindawiya	Dec 2020–Jan 2021 with Feb 2021–Jun 2021	0.02
Al-Rawabi	Dec 2020–Jan 2021 with Feb 2021–Jun 2021	0.15
Al-Safa	Dec 2020–Jan 2021 with Feb 2021–Jun 2021	0.39
As-Salamah	Dec 2020–Jan 2021 with Feb 2021–Jun 2021	0.29
As-Salamah	Feb 2021–Jun 2021 with Dec 2021–Jan 2022	–0.08
As-Salamah	Dec 2021–Jan 2022 with Feb 2022–Apr 2022	0.20

**Table A8:** Correlation in modelled mosquito presence surfaces for trap collection locations.

District	Comparison	Kendall’s Rank Correlation
Al-Hindawiya	Dec 2020–Jan 2021 with Feb 2021–Jun 2021	–0.34
Al-Rawabi	Dec 2020–Jan 2021 with Feb 2021–Jun 2021	–0.05
Al-Safa	Dec 2020–Jan 2021 with Feb 2021–Jun 2021	–0.34
As-Salamah	Dec 2020–Jan 2021 with Feb 2021–Jun 2021	–0.01
As-Salamah	Feb 2021–Jun 2021 with Dec 2021–Jan 2022	–0.07
As-Salamah	Dec 2021–Jan 2022 with Feb 2022–Apr 2022	0.02
